# Impedimetric DNA Sensor Based on Electropolymerized N-Phenylaminophenothiazine and Thiacalix[4]arene Tetraacids for Doxorubicin Determination

**DOI:** 10.3390/bios13050513

**Published:** 2023-04-30

**Authors:** Tatjana Kulikova, Igor Shiabiev, Pavel Padnya, Alexey Rogov, Gennady Evtugyn, Ivan Stoikov, Anna Porfireva

**Affiliations:** 1 A.M. Butlerov’ Chemistry Institute of Kazan Federal University, 18 Kremlevskaya Street, 420008 Kazan, Russia; wefy2009@yandex.ru (T.K.); shiabiev.ig@yandex.ru (I.S.); pavel.padnja@kpfu.ru (P.P.); gennady.evtugyn@kpfu.ru (G.E.); ivan.stoikov@mail.ru (I.S.); 2 Interdisciplinary Center of Analytical Microscopy of Kazan Federal University, 18 Kremlevskaya Street, 420008 Kazan, Russia; 1arogov@kpfu.ru; 3 Analytical Chemistry Department, Chemical Technology Institute, Ural Federal University, 19 Mira Street, 620002 Ekaterinburg, Russia

**Keywords:** electropolymerization, phenothiazine derivative, thiacalix[4]arene acid, electrochemical impedance spectroscopy, electrochemical DNA sensor, doxorubicin determination

## Abstract

Electrochemical DNA sensors are highly demanded for fast and reliable determination of antitumor drugs and chemotherapy monitoring. In this work, an impedimetric DNA sensor has been developed on the base of a phenylamino derivative of phenothiazine (PhTz). A glassy carbon electrode was covered with electrodeposited product of PhTz oxidation obtained through multiple scans of the potential. The addition of thiacalix[4]arene derivatives bearing four terminal carboxylic groups in the substituents of the lower rim improved the conditions of electropolymerization and affected the performance of the electrochemical sensor depending on the configuration of the macrocyclic core and molar ratio with PhTz molecules in the reaction medium. Following that, the deposition of DNA by physical adsorption was confirmed by atomic force microscopy and electrochemical impedance spectroscopy. The redox properties of the surface layer obtained changed the electron transfer resistance in the presence of doxorubicin due to its intercalating DNA helix and influencing charge distribution on the electrode interface. This made it possible to determine 3 pM–1 nM doxorubicin in 20 min incubation (limit of detection 1.0 pM). The DNA sensor developed was tested on a bovine serum protein solution, Ringer–Locke’s solution mimicking plasma electrolytes and commercial medication (doxorubicin-LANS) and showed a satisfactory recovery rate of 90–105%. The sensor could find applications in pharmacy and medical diagnostics for the assessment of drugs able to specifically bind to DNA.

## 1. Introduction

Electropolymerization is a unique approach to developing modifying layers in biosensor design. There are many different electropolymerized coatings which exert their own redox activity and take part in electron transfer within the layer and on the electrode interface. Pyrrole [1], aniline [2], thiophene [3] and their derivatives [4,5,6] are the most popular precursors that are electrochemically polymerized and show electroconductive properties. The performance of electropolymerized layers depends on the monomer structure, pH, electrodeposition conditions and electrolyte content of the reaction media.

Phenazine, phenoxazine and phenothiazine derivatives are another type of species able to form electrochemically active films via electrolysis [7,8,9]. They became widespread due to their ability to mediate electron transfer and electrostatically accumulate many analytes and biomolecules applied in biosensor assembling. Similarly to polyaniline, which is mostly applied as a support for biocomponents [10,11], electropolymerized phenothiazine and phenazine dyes offer good opportunities for electric wiring of binding sites and have found increasing application in immuno- and DNA sensors [12,13,14]. Their use is also promoted by milder conditions of electropolymerization against those of polyaniline. They can be electrodeposited onto an electrode in neutral media. As opposed to polyaniline, there is no necessity to electrodeposit these monomers from acidic media, which is their essential advantage when working with biorecognition agents, e.g., DNA.

The electrochemical characteristics of methylene blue as a precursor of the supporting layer were studied in [15], where electrochemical deposition of the polymeric form was performed onto an electrode modified with multi-walled carbon nanotubes in an aqueous solution. In addition, poly(methylene blue) layers have been obtained on electrodes preliminarily modified with ZnO nanoparticles [16], copper-carbon nanofibers [17] and NiO nanoflowers [18].

Redox mediation activity of poly(methylene green) was shown by Tsuruoka et al. [19]. They grafted poly(methylene green) onto porous carbon electrodes and obtained high currents of glucose oxidation. Electropolymerization of proflavine was performed on a glassy carbon electrode (GCE) [20]. The modified electrode was then utilized for immobilization of DNA and detection of the DNA intercalators. Cyclic voltammetry, scanning electron microscopy and impedance spectroscopy were used to characterize the redox properties of proflavine polymer prior to and after DNA deposition. The limits of detection (LOD) were 0.3 and 0.001 nM for doxorubicin and daunorubicin, respectively.

Liu et al. applied electropolymerized polythionine for GCE modification followed by DNA immobilization [21]. Due to negative charge of the phosphate groups in the DNA backbone, positively charged polythionine layer promoted accumulation of the DNA molecules on the electrode interface. After that, Ag nanoparticles bearing a positive charge were deposited on the surface. The construction technique applied was utilized for the immobilization of horseradish peroxidase and hydrogen peroxide determination with good sensitivity and acceptable stability of the signal. Other examples of polythionine being incorporated into DNA sensors involve the detection of Cd in mussels with an aptasensor based on polythionine–Au nanoparticles [22] and electrochemiluminescent detection of hybridization events [23].

The synthesis and characterization of novel polythiophene derivatives containing porphyrin units were reported for future possible application in solar cells [24]. Their electrochemical behavior was studied by cyclic voltammetry in organic media, where they showed lower oxidation potential in comparison with polythiophene.

The electropolymerization of phenazine dyes presents complications related to the pH dependence of redox properties, monomer aggregation and its low solubility in aqueous media. Aggregation can accelerate charge-recombination processes and make worse the reproducibility of the surface layer characteristics and reversibility of the electron transfer. In this regard, it is important to extend the variety of phenothiazine derivatives and characterize their performance in electropolymerization and DNA sensor assembling. Recently, we have described the electrochemical properties of *N*-phenyl-3-(phenylimino)-3H-phenothiazin-7-amine (PhTz, Figure S1 of Electronic Supporting Information, ESI, Supplementary Materials) [25].

It was shown that PhTz in its monomeric form is involved in the quasi-reversible process of electron transfer, which is affected by DNA deposition due to electrostatic interactions. Meanwhile dense contact between the DNA backbone and polymeric film can sterically hinder the access of small molecules able to biospecific interactions with DNA. Previously we have shown that the incorporation of charged macrocycles able to multiply non-covalent interactions with DNA alters the morphology of the surface layer and enhances the electrochemical response related to the detection of DNA damage and anticancer drug determination [26,27,28].

In this work, the thiacalix[4]arene carboxylic acids in various configurations bearing four carboxylate groups have been for the first time applied in the assembling of DNA-sensing layers and the impedimetric determination of doxorubicin as a model DNA intercalator.

## 2. Materials and Methods

### 2.1. Reagents

The phenothiazine derivative PhTz was synthesized as described in [29], and thiacalix[4]arene tetracarboxylic acids (Figure S2) were synthesized as described in [30] at the Organic and Medicinal Chemistry Department of Kazan Federal University.

HEPES (4-(2-hydroxyethyl)-1-piperazineethanesulfonic acid) and DNA from fish sperm and salmon testes were purchased from Sigma-Aldrich (Dortmund, Germany). Doxorubicin-LANS^®^ (“Verofarm”, Moscow, Russia) was purchased from a local pharmacy. All other reagents were of analytical grade and applied without further purification. Working 0.05 M phosphate buffer and 0.1 M HEPES, used in electropolymerization and electrochemical investigations, respectively, contained 0.1 M NaNO_3_ as a supporting electrolyte. Millipore^®^ water (Simplicity^®^ Water Purification System, Merck-Millipore, Mosheim, France) was used for the preparation of working solutions for all the measurements. Voltammetric and impedimetric measurements were carried out in presence of a 0.01 M mixture of K_3_[Fe(CN)_6_] and K_4_[Fe(CN)_6_]. For electrochemical measurements and electropolymerization, PhTz was dissolved in acetone and then mixed with phosphate buffer (pH = 7.1) at a 1:1 (*v*/*v*) ratio. The pH of the obtained mixture was additionally adjusted by NaOH/HCl to the requested pH prior to measurements. For assessment of the possible influence of serum electrolytes, Ringer–Locke’s solution (0.45 g NaCl, 0.021 g KCl, 0.016 g CaCl_2_·2H_2_O, 0.005 g NaHCO_3_, 0.015 g of MgSO_4_ and 0.025 g NaH_2_PO_4_·2H_2_O in 50 mL of deionized water [31]) was used.

### 2.2. Apparatus

Voltammetric and impedimetric measurements were performed with an Autolab PGSTAT302 N potentiostat/galvanostat equipped with the FRA32M module (Metrohm Autolab b.v., Utrecht, The Netherlands) at room temperature in a three-electrode cell. GCE (2 mm in diameter, OhmLiberScience, Saint-Petersburg, Russia) was applied as the working electrode; Ag/AgCl (3.0 M NaCl) (Metrohm Autolab b.v. Cat No 6.0733.100) as the reference electrode; and Pt wire as the counter electrode.

Electrochemical impedance spectra (the Nyquist diagrams) were recorded at the frequency range from 100 kHz to 0.04 Hz with an amplitude of 5 mV. The impedance parameters were determined by fitting with the Randles equivalent circuit [*R_s_*(*Q*[*R_et_W*])], where *R_s_* is the solution resistance, *Q* the constant-phase element (CPE), *R_et_* the electron transfer resistance and *W* the Warburg element. Equivalent circuit fitting was performed with the NOVA software (Metrohm Autolab b.v.).

Atomic force spectroscopy (AFM) images were obtained with a Dimension FastScan probe microscope (Bruker, Germany) in the quantitative nanomechanical mapping mode with “Bruker ScanAsyst Air” silicon probes (curvature radius ~2 nm) and *k* 0.4 N/m. The scan rate was equal to 1 Hz within a 256 × 256 window. Image processing was performed with the Gwyddion-Free SPM (version 2.57) data analysis software.

### 2.3. Electrode Modification

GCE was first mechanically polished with 0.05 µm alumina powder and then washed with deionized water. After that, it was immersed in a mixture of 0.2 M sulfuric acid and acetone (1:1 *v*/*v*), and its potential was cycled between −0.5 and 1.0 V until stabilization of the voltammogram. After that, it was washed again with buffer and acetone and dried at room temperature. For PhTz electropolymerization, the electrode was moved to 3 mL of solution containing 1.5 mM 50 mM phosphate buffer and 100 mM NaNO_3_ mixed with 1.33 mL of acetone. After stabilization by running 10 cycles of the potential between −0.1 and 1.0 V (100 mV/s), 170 µL of 0.5 mg/mL pf PhTz in acetone was added to the same solution, and the potential was scanned in the same potential range for 2.5 cycles. The polymerization product was additionally stabilized by moving the electrode to the HEPES buffer with no PhTz monomer and running one additional potential cycle in the same conditions. DNA if mentioned was drop-casted onto the electrode surface with electropolymerized PhTz, left for 20 min for adsorption and then washed with working buffer. Implementation of thiacalix[4]arene carboxylic acids in the surface layer was performed by their addition to the solution containing monomeric PhTz at a molar ratio of 1:1, 1:2 or 1:4.

### 2.4. Doxoruibcin Determination

The DNA sensor assembled as described above was fixed upside down. An aliquot of doxorubicin solution or spiked serum sample was drop-casted onto the surface, and the sensor was covered with a plastic tube to prevent drying of the solution. After incubation, the electrode was washed with deionized water and working buffer, and the EIS spectra were recorded in 0.01 M [Fe(CN)_6_]^3−/4−^ solution. In a blank experiment, the DNA sensors were exposed in a similar manner in deionized water and buffer solution with no doxorubicin. In addition, similar experiments were performed with GCE covered with polyPhTz with no DNA.

## 3. Results

### 3.1. Polymerization of PhTz and Cyclic Voltammetry of the Surface Layer Obtained

**Figure 1:**
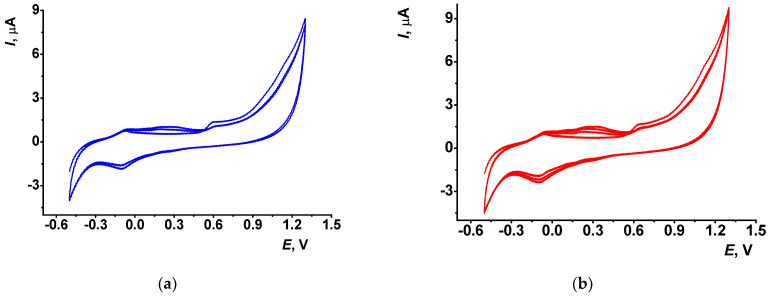
Cyclic voltammograms of PhTz polymerization recorded in phosphate buffer–acetone mixture (1:1 *v*/*v*) in the presence of 0.072 mM PhTz, scan rate 100 mV/s: (**a**) 2.5; (**b**) 3.5 cycles of potential.

Previously, we have studied electrochemical behavior of monomeric PhTz [25] and showed the reversible redox conversion of the phenothiazine core of the molecule. In this work, we started from the consideration of the conditions of the polyPhTz electrodeposition on bare GCE. The coating expected should be as thin as possible but cover all of the surface of the working electrode. For aniline electropolymerization, it was shown that two to three cycles of potential scanning were sufficient to obtain the polymer film [32]. Figure 1 shows that cyclic voltammograms recorded on GCE in the solution contained 0.072 mM PhTz. Electropolymerization with a higher number of potential scans was considered in [25].

It is obvious that such a limited number of cycles cannot result in the formation of large molecular products of electrolysis. In the following discussion, electropolymerization products are rather assigned to oligomers insoluble in working media and deposited on the electrode interface.

The electropolymerization is initiated by irreversible oxidation of the PhTz molecule at high anodic potential (1.3 V). When the potential scan was reversed at lower potential, no changes in the following scans on voltammograms were found. With multiple cycling, a new pair of redox peaks appeared and grew at 0.30–0.35 V, which testifies the deposition of the polymeric form of PhTz. After the first three to four scans, the above peaks regularly increased, but after the fifth cycle (not shown) the peaks were stabilized and became much broader, indicating a limitation of the monomer access to the electrode surface and a slower electron transfer.

A half-cycle means the final potential was defined as the highest anodic potential to accumulate the most oxidized (and positively charged) form of the polymerized PhTz favorable from the point of view of electrostatic interactions with DNA and thiacalix[4]arene tetracarboxylic acids tested as layer components.

**Figure 2:**
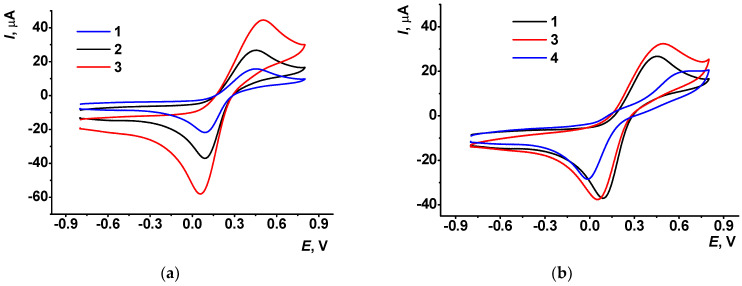
Cyclic voltammograms recorded in 0.01 M [Fe(CN)_6_]^3−/4−^ solution on GCE (**a**), bare (1) and that covered with the polyPhTz synthesized in 2.5 (2) and 3.5 (3) potential cycles; (**b**) polyPhTz synthesized in the presence of TC-*cone* (3) and that after 20 min incubation of DNA (4) (2.5 cycles).

The GCE modified with polyPhTz transferred in the buffer with no monomer did not show remarkable peaks of the monomer but demonstrated better conditions of electron exchange. It was confirmed by recording [Fe(CN)_6_]^3−/4−^ voltammograms (Figure 2).

The peak potential difference (0.36 V for 2.5 cycles and 0.42 V for 3.5 cycles) and the peak current ratio (*I_pa_*/*I_pc_* = 1.1 for 2.5 cycles and 1.0 for 3.5 cycles of the potential scan) were typical for quasi-reversible electron transfer (Figure 2a). Meanwhile the peak currents increased with the number of potential scans, indicating the contribution of the polymer to the electron transfer. The equilibrium potential calculated as a half-sum of appropriate peak potentials of the [Fe(CN)_6_]^3−/4−^ pair was about 0.27 V for both coatings (2.5 and 3.5 cycles). It is shifted against that recorded on bare GCE (0.23 V) to more positive values. It can be concluded that the deposition of the polyPhTz layer did not interfere with the electron exchange reaction of ferricyanide ions, and this redox probe can be further used in the EIS measurements.

The addition of thiacalix[4]arene carboxylic tetraacids (named hereafter as macrocyclic acids) to the reaction media surprisingly increased the currents related to the accumulation of the polyPhTz form on the GCE (Figure S3) and the ferrocyanide oxidation peak on the voltammogram (Figure 2b). Being electrochemically inactive, macrocyclic acids promoted adsorption of positively charged electropolymerization products on the electrode. The influence is more pronounced on the direct (anodic) branch of the cyclic voltammograms, whereas the cathodic peak currents on the reversed branch are about the same with no respect of the presence of the macrocycle in the solution. The concentration of macrocyclic acids was selected to be 1:1, 1:2 and 1:4 mol/mol against PhTz to control the charge of the complex formed in electrostatic interactions. Assuming the presence of the PhTz units in the polymer as dications and full dissociation of macrocyclic acids into tetraanions, the 1:1 ratio corresponds to a negative charge of the 1:1 complex, 1:2 ratio to a neutral complex and 1:4 ratio to a positively charged complex of PhTz and the macrocycles studied.

The influence of the configuration of the macrocycle (denoted as TC-*cone* for *cone*, TC-*paco* for *partial cone* and TC-*alt* for *1,3-alternate*, see Figure S2 for chemical structures) on the shape and relative position of the[Fe(CN)_6_]^3−/4−^ peak pair was found to be insignificant. From this fact, the influence of the macrocycles tested can be attributed rather to the total charge of the reactants at the stage of their transfer to the electrode than to the spatial limitations resulting from the absolute volume of the counterparts.

Adsorption of the DNA molecules on the polyPhTz–thiacalixarene layer decreased the ferri-/ferrocyanide peak currents and increased the peak potential difference due to the incorporation of large non-conductive biomolecules in the surface film. Their charge is opposite to that of the redox probe and affects the access of the redox probe. The comparison of the influence of two anionic species (macrocyclic acids vs. DNA) on the conditions of electron transfer in the polyPhTz layer made it possible to conclude that incorporation of thiacalix[4]arene carboxylic acids is governed by electrostatic forces and does not result in changing the charge of the layer–solution interface. DNA molecules adsorbed on the surface of the electrodeposited product provide negative charge to the interface and hence prevent access by ferri-/ferrocyanide anions.

### 3.2. AFM Measurements

**Figure 3:**
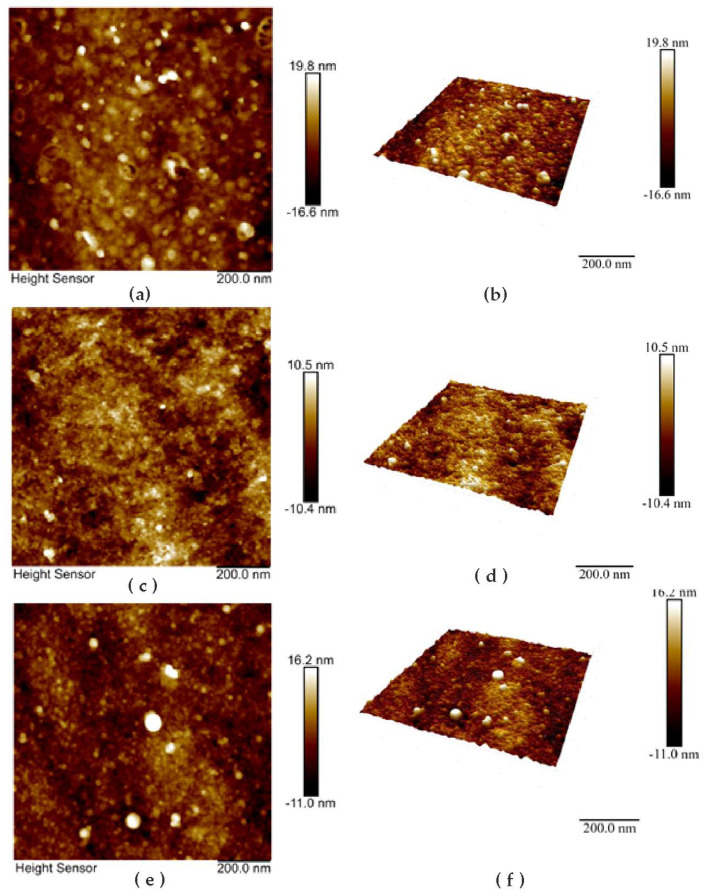
Morphology (**a**,**c**,**e**) and 3D models of the GCE surface (**b**,**d**,**f**) after electrodeposition of polyPhTz (**a**,**b**); polyPhTz in the presence of TC-*cone* (**c**,**d**); and that after 20 min exposition of 1 mg/mL DNA (**e**,**f**).

**Figure 4:**
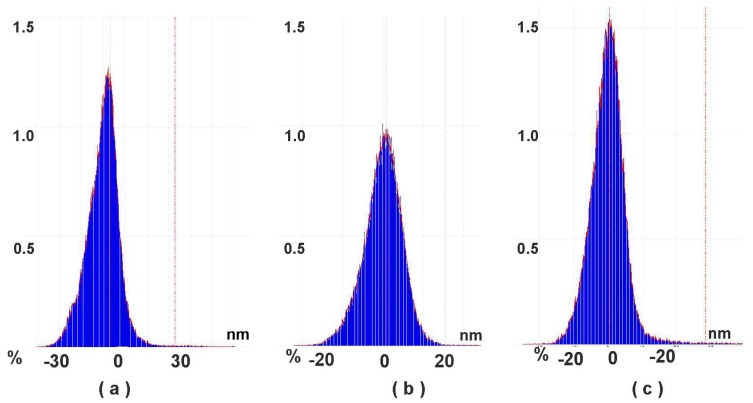
Particle size distribution calculated from AFM data: (**a**) GCE—polyPhTz (2.5 cycles of electropolymerization); (**b**) GCE—polyPhTz/TC-*cone*; (**c**) GCE—polyPhTz/TC-*cone*—DNA.

In accordance with the AFM data, electropolymerization of PhTz results in the formation of irregular roundish particles evenly distributed along the electrode surface. The AFM images and 3D models of the surface morphology are presented in Figure 3 and the particle size distribution in Figure 4. In 2.5 cycles of potential scanning, some particles had a toroid form with a small cavity in the central part. In 3.5 cycles of polymerization, the number of particles increased, and the maximal profile difference became slightly lower. The addition of macrocyclic acids to the PhTz solution resulted in the formation of a more regular coating with a narrower size distribution. The relief difference decreased about twice over. The maximum on the size distribution histogram (Figure 4b) also decreased about twice over compared to the PhTz coating. Probably the PhTz–TC interaction resulted in the disaggregation of the phenothiazine molecules and the acceleration of their coupling in the electrochemical step. As a result, the rate of deposition increased, and the average size of the polymer seeds decreased against similar characteristics obtained in the PhTz polymerization in the absence of the macrocyclic acid (Figure 4a vs. Figure 4b). It should be noted that the concentration chosen for the PhTz monomer was near the maximum available in the aqueous medium. Low solubility of the PhTz was the reason to add 50% acetone to the buffer applied in the electropolymerization step. This explains the variety of the polymer particles formed in conditions of low solubility and random aggregation. Macrocyclic acids promote the disappearance of the lowest particles that either increase faster in size or amalgamate via bridging macrocycle molecules with the formation of supramolecular associates. The ensuing adsorption of the DNA molecules did not alter the overall size variation of the particles but increased the percentage of the median size (compare Figure 4b,c). This means the DNA did not affect the granulation of the PhTz–TC-*cone* particles, but its adsorption increased the size of appropriate granules. Thus, AFM data confirmed the suggestion about the mechanism of the influence of macrocyclic acids on PhTz polymerization and the electrochemical properties of the product.

### 3.3. EIS Measurements

**Figure 5:**
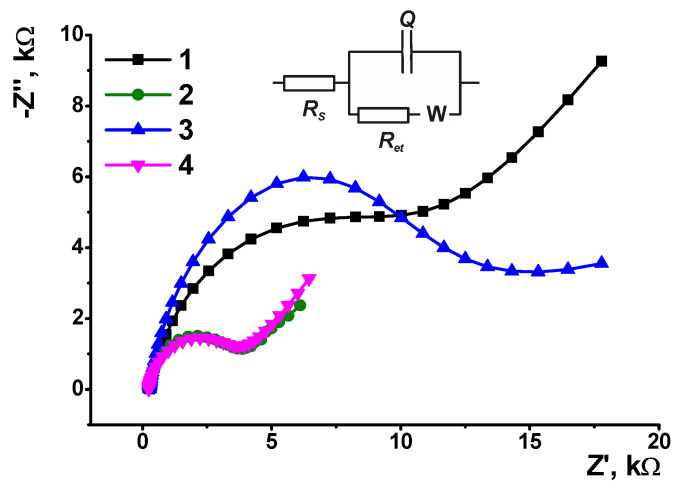
The Nyquist diagrams obtained with GCE covered with polyPhTz (2.5 cycles) in the presence of TC-*cone*, reagent ratio TC-*cone*: PhTz (mol/mol) = 0 (1), 1:4 (2), 1:2 (3) and 1:1 (4). Inset: equivalent circuit, *R_s_*—solvent resistance, *R_et_*—electron transfer resistance, *W*—Warburg impedance, *Q*—constant phaser element.

EIS parameters were calculated from the fitting data with the *R*(*Q*[*RW*]) equivalent circuit (Randles circuit). EIS measurements were performed in the presence of a 0.01 M [Fe(CN)_6_]^3−/4−^ redox probe. Preliminary investigations performed in cyclic voltammetry mode showed no influence of interlayer electron exchange on the [Fe(CN)_6_]^3−/4−^ redox behavior. The Nyquist diagram contained a semicircle in the area of high frequencies corresponding to the step electron transfer as a limiting step of electrode reaction and a linear part attributed to the diffusion control. The diameter of the semicircle corresponds to the electron transfer resistance (*R_et_*) and is commonly used for assessment of the surface layer permeability and electron exchange conditions on the electrode interface. All the calculations were conducted for three individual electrodes prepared with the same set of reagents; GCE covered with polyPhTz (2.5 cycles) showed an *R_et_* value of 10.9 ± 0.5 kΩ because the polymer deposited showed redox activity but not electroconductivity and hence limited the rate of electron exchange on the electrode interface. In agreement with this statement, the 3.5 scans of the potential increased the *R_et_* value to 18.9 ± 2.3 kΩ. The accumulation of the charged polymer resulted in an increase of the constant phase element *Q* from 8.9 ± 1.4 μF to 15.2 ± 0.8 μF. *Q* was interpreted here as capacitance because the roughness coefficient was near 0.9 for all the EIS measurements discussed in this section. The addition of macrocyclic acids to the reaction medium alters the EIS parameters (Figure 5).

As can be seen, the use of charged complexes formed by macrocyclic acids and PhTz (1:1 and 1:4 molar ratio of reagents) resulted in similar decreases of the *R_et_* values (3.3 ± 0.7 and 4.1 ± 0.5 kΩ, respectively), whereas the use of a neutral complex with two PhTz units per one tetracarboxylic acid derivative (1:2 ratio) gave the opposite result (*R_et_* = 14.6 ± 1.4 kΩ against 10.9 ± 0.5 kΩ for the GCE/polyPhTz electrode). Probably, charged species are disaggregated more effectively, and this influences the efficiency of polymerization to a greater extent than the implementation of non-conductive and electrochemically inactive counterparts. The capacitance of the electrode described was found to be insignificant to the macrocyclic acid added. In all the coatings, it was equal to 9–11 μF.

The results of PhTz electropolymerization depended on the configuration of the macrocyclic acid. Thus, for TC-*alt*, the *R_et_* changed in the range of molar ratios 1:4, 1:2 and 1:1 from 2.8 ± 0.3 to 15.2 ± 0.7 kΩ and 0.56 ± 0.03 kΩ, respectively. Contrary to that, the capacitance changed in the opposite direction (7.8 ± 0.6, 12.3 ± 0.7 and 112 ± 5 μF, respectively). It should be noted that in the *1,3-alternate* configuration, the carboxylic groups are located on the opposite sides of the plane of the macrocycle core (see Figure 2) so that interaction with a flat phenothiazine fragment of the polymer does not result in full neutralization of the negative charge and leaves open the possibility of further electrostatic interactions. This offers more possibilities regarding the assembly redox active layer on the electrode surface and creates more favorable conditions for electrodeposition of the 1:1 complex with a total positive charge against those with negative charges of the interface. Thus, the significantly lower *R_et_* value calculated for TC-*alt* can be attributed to the electrostatic attraction of negatively charged ferri-/ferrocyanide ions used in EIS measurements as redox probes. The *partial cone* configuration (TC-*paco*) did not show significant differences from the behavior of TC-*cone* and demonstrated the same *R_et_* values for the 1:4 and 1:1 complexes.

At the next step, the DNA solution was drop-casted onto the modified electrode and left for a certain period of time for equalization and adsorption. A 10 min incubation led to a 2.5-fold increase of the *R_et_* value due to recharging of the electrode surface and electrostatic repulsion of ferri-/ferrocyanide anions. Oppositely, when a thicker coating (3.5 cycles of potential scanning) was assembled with the same DNA quantity, the electron transfer resistance value decreased twice over against GCE/polyPhTz (not shown). To improve the sensitivity of the response toward DNA loading, the incubation period was extended to 20 min. The following increase of exposition did not shift the EIS parameters confirming equalization of the surface layer content but increased their deviation in a series of individual sensors (sensor-to-sensor repeatability higher than 15%).

**Figure 6:**
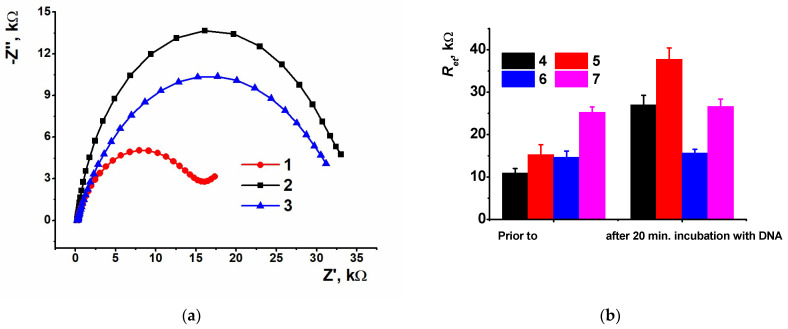
(**a**) The Nyquist diagrams obtained with GCE covered with polyPhTz (2.5 cycles) in the presence of TC-*cone* (1), TC-*paco* (2) and TC-alt (3) and coated with DNA (20 min incubation, 1 mg/mL of DNA from fish sperm); (**b**) comparison of the electron transfer resistance for various coatings of the GCE/PolyPhTz/DNA sensors prior to and after DNA loading: polyPhTz (4), poly-PhTz TC-*alt* (5), polyPhTz—TC-*cone* (6), polyPhTz—TC-*paco* (7).

Figure 6 shows the Nyquist diagrams corresponding to the equal loading of DNA (1 mg/mL of DNA from fish sperm) and incubation period (20 min) on the GCE/polyPhTz sensor obtained in the presence of various macrocyclic acids.

As can be seen, the DNA deposition discriminated the *R_et_* values related to various macrocyclic acids. The greatest difference was found for *1,3-alternate*, confirming the effect of the symmetrical positions of carboxylate groups and non-compensated negative charge of the complex. The *cone* configuration with the carboxylate groups directed to one side of the macrocycle plane is shown lower. The substitution of the DNA from fish sperm with that from salmon testes did not affect the EIS parameters.

### 3.4. Doxorubicin Determination

Doxorubicin is a chemotherapy medication that belongs to the anthracycline family widely used in treating lung, thyroid, ovarian, gastric and especially breast cancer [33,34]. Despite high efficiency, doxorubicin exerts severe adverse effects, e.g., cardiotoxicity, therapy related malignancies and gonadotoxicity [35]. For this reason, it is important to monitor both the doxorubicin level in biological fluids and its pharmacokinetics in chemotherapy. At present, doxorubicin levels are determined by UV–vis spectrometry [36], chromatography [37,38], fluorimetry [39], capillary electrophoresis [40] and chemiluminescence [41]. Being rather sensitive, such techniques are rather expensive, require complicated maintenance and are labor- and time-consuming. Electrochemical DNA sensors offer an alternative to conventional instrumentation for preliminary control of doxorubicin concentration compatible with a point-of-care texting format.

#### 3.4.1. Measurement Conditions

Doxorubicin as a DNA intercalator is included between pairs of complementary nucleobases of double-stranded DNA. This can lead to changes in the specific volume and flexibility of DNA strands, often resulting in further DNA damage in reactions with reactive oxygen species. Structural DNA changes can be electrochemically monitored if the DNA molecules are immobilized on a surface with redox properties sensitive to charge variation.

**Figure 7:**
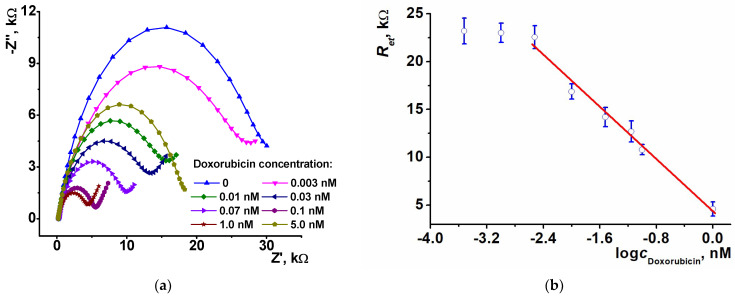
(**a**) The Nyquist diagrams obtained with GCE covered with polyPhTz (2.5 cycles) in the presence of TC-*alt* and DNA (20 min incubation, 1 mg/mL of DNA from salmon testes) after incubation in increasing concentrations of doxorubicin (20 min. incubation; (**b**) calibration graph of doxorubicin determination in semilogarithmic plots, average ± S.D. for three individual sensors.

The coating based on electropolymerized PhTz (2.5 cycles) in the presence of thiacalix[4]arene derivative in the *1,3 alternate* configuration (TC-*alt*) was selected for doxorubicin determination. DNA from salmon testes was applied to the polymer surface. In comparison with that from fish sperm, it contains more double-stranded DNA fragments [42] and hence should be more sensitive to intercalators. In the concentration range from 0.003 to 1 nM, the electron transfer resistance regularly decreased with the drug concentration (Figure 7).

At higher doxorubicin concentration, the direction of the changes reversed and became irreproducible, probably due to desorption of intercalated DNA from the polymer layer. This might result from rather weak binding forces in supramolecular aggregates formed on the electrode interface. When an intercalator penetrates the double-stranded DNA helix, the electrostatic interactions acting as film-forming factor become weaker. First, DNA increases the diffusional barrier of the ferri/ferrocyanide indicator access, but then the density of the barrier changes irregularly, and the deviation of the resulting response grows.

The response toward doxorubicin is probably due to partial separation of negative charge centers in the DNA duplex and the decrease of the specific charge on the polymeric layer. Overloading of the DNA molecules can result in neutralization of the charge by a protonated amino group of a doxorubicin molecule.

The signal of the sensor is linearized in semilogarithmic plots in accordance with Equation (1) (n is the number of experimental points within the linear range of the calibration curve).

*R_et_*, kΩ = (4.3 ± 0.5) − (6.84 ± 0.44) × log(*c*_Dox_, nM), *n* = 6, *R*^2^ = 0.985 (1)

The detection limit (LOD) assessed from an S/N = 3 ratio was equal to 1.0 pM. The limit of quantification was calculated from an S/N = 10 ratio and was 3.0 pM. The comparison with the performance of other electrochemical sensors for doxorubicin determination is presented in Table 1. One can see the impedimetric sensor proposed allowed high sensitivity and low concentrations determined against other sensors described. The only exception is the detection of DNA-specific interactions based on the use of ultra-thin polyaniline films [32] and acridine yellow monomer adsorbed on the electrode [43], where the electrostatic influence of doxorubicin binding is amplified by changes in the electroconductive properties of polyaniline and by desorption of the dye molecules from the electrode interface.

#### 3.4.2. Measurement Precision and Lifetime

Sensor-to-sensor repeatability was calculated from the response of 6 individual sensors to 0.1 nM doxorubicin (20 min incubation). For freshly prepared sensors the RSD was equal to 4.5% and increased to 7.2% within a day when placed between the measurements in the working buffer. In dry conditions the sensor retained its sensitivity toward doxorubicin for at least two weeks when stored in dry conditions at 4 °C. The lifetime can be sufficiently extended if the DNA loading is directly performed prior to exposition in doxorubicin solution. In such a format, the RSD. for the sensors stored for six weeks was 5.5%. All the sensors contacted the doxorubicin only once, and attempts to recover sensors after signal measurement resulted in irreproducible changes of the EIS parameters.

#### 3.4.3. Selectivity and Real Sample Assay

Doxorubicin’s influence on the redox properties of the polyPhTz layer assumed intercalation of the DNA molecules so that other medications with similar effects would interfere with doxorubicin for DNA binding. This can be related to antitumor drugs with structures similar to that of doxorubicin (daunorubicin, idarubicin, valrubicin, etc.). Thus, we have shown previously the possibility for determination of daunorubicin ([20], LOD of daunorubicin 1.0 pM, poly(proflavine) as a DNA matrix) and idarubicin ([43], LOD 0.3 fM, poly(Azure A) as a DNA matrix) based on a similar mechanism of signal generation (changes in the polymer redox properties due to intercalation process). Although the concentrations detected vary, all of the anthracycline drugs affected the response measured in direct-current voltammetry or EIS mode. Higher sensitivity of idarubicin determination was reached due to the application of methylene blue as an auxiliary mediator of electron transfer. In addition, all three anthracycline medications mentioned were compared in the same measurement conditions with a polyaniline-DNA biosensor ([56], LODs of 0.01 nM doxorubicin, 0.1 nM daunorubicin and 0.2 nM idarubicin). It should be noted that anthracycline drugs are mostly applied separately, and medications differ in the nature of auxiliary components providing target delivery of the substances to the solid tumor (lipids, stabilizers, etc.). Meanwhile, sulfonamide preparations were shown to be rather inert and did not affect the signal of anthracyclines measured with similar sensors with electropolymerized coatings [56].

Bovine serum albumin was added to the HEPES buffer as a model of serum proteins and Ringer–Locke’s solution (see content in Experimental section) as a model of plasma electrolytes. The recoveries of the detection of 0.1 nM doxorubicin were 110 ± 15% and 95 ± 10%, respectively.

Doxorubicin-LANS^®^ (“Verofarm”) was dissolved in 0.1 M HEPES and used for the incubation of the impedimetric sensor as described above for a standard solution of the drug. The recovery was calculated using a calibration plot for a 0.1 nM nominal concentration, and a recovery of 92 ± 12% was found for three individual sensors.

## 4. Discussion

The formation of ultrathin layers of a phenylamino derivative of phenothiazine, PhTz, in neutral solution made it possible to obtain on bare GCE a stable film with mediation activity and affinity toward DNA molecules specifically adsorbed on the film by drop-casting protocol. Contrary to previously described investigations of the electrochemical properties of PhTz, the electrodeposition of the redox-active layer was performed with no additional modifiers like carbon black in one step. The addition of carboxylic derivatives of thiacalix[4]arene bearing four carboxylate groups promoted the deposition of electroactive products by electrostatic interactions. The resulting redox activity of the polymer coating and its interaction with DNA depended on the configuration of macrocyclic acids, and contribution was maximal for the symmetrical *1,3-alternate* derivative. The molar ratio of macrocyclic acids and PhTz monomer in the reaction medium was found to be most important for the redox properties of the sensor and its applicability for the detection of biospecific interactions of DNA. The influence of both DNA and macrocyclic acids was attributed to electrostatic interactions and the formation of the differently charged complexes affecting the access of the redox probe ([Fe(CN)_6_]^3−/4−^) to the electrode. The intercalation of DNA with doxorubicin diminishes the charge of phosphate residues because of the shielding effect and neutralization of the charge with the amino group of the drug molecules. This resulted in changes in the electron transfer resistance. The signal was found to be quite stable and sensitive, including in measurements in the presence of serum albumin and plasma electrolytes.

The simple design of the sensing layer and the high sensitivity of doxorubicin determination make the impedimetric sensor developed attractive for new antitumor drug selection, pharmacokinetics and chemotherapy monitoring of oncology patients [57].

## Figures and Tables

**Table 1:** Analytical characteristics of the determination of doxorubicin with electrochemical sensors and DNA sensors.

Modifier	Concentration Range	LOD, nM	Ref.
Electrochemical sensors			
Mesoporous carbon nanospheres/rGO	10 nM–10 μM	1.5	[44]
Pyrographite	10 nM–1.0 μM	10	[45]
Silver amalgam	0.6–10 μM	440	[46]
/MgO/carbon nanodots/	0.1–1.0 μM	90	[47]
Tryptophan/PEG/CoFe_2_O_4_	60 nM–2.0 μM	30	[48]
Electrochemical DNA sensors			
poly(Azure B)	0.1 nM–0.1 μM	0.07	[49]
Polyaniline	1.0 pM–1000 μM	0.0006	[32]
CNTs–polylysine	2.5 nM–0.25 μM	1.0	[50]
Pt/Ag nanoparticles	0.2–2.0 μM	-	[51]
SWCNTs	1. nM–20 μM	0.6	[52]
BDD/DNA aptamer	Up to 2.3 μM	49	[53]
Copolymer of Azure A and proflavine	0.03–10 nM	0.01	[54]
Acridine Yellow adsorbed on GCE	0.1 pM–1.0 nM	0.0007	[55]
PolyPhTz/macrocyclic acids	3.0 pM–1.0 nM	0.001	This work

Acronyms: rGO—reduced graphene oxide, PEG—poly(ethylene glycol), CNTs—carbon nanotubes, SWCNTs—single-wall carbon nanotubes, BDD—boron-doped diamond.

## Data Availability

Data is contained within the article and supplementary material.

## Supplementary Materials

The following supporting information can be downloaded at: https://www.mdpi.com/article/10.3390/bios13050513/s1, Figure S1: Chemical structure of *N*-Phenyl-3-(phenylimino)-3H-phenothiazin-7-amine studied; Figure S2: Chemical structures of thiacalix[4]arene tetracarboxylic acids studied; Figure S3: Cyclic voltammograms of PhTz polymerization recorded in phosphate buffer–acetone mixture (1:1 *v*/*v*) in the presence of 0.072 mM PhTz and TC-*cone*.
