# Supplementary material for: Impedimetric DNA Sensor Based on Electropolymerized N-Phenylaminophenothiazine and Thiacalix[4]arene Tetraacids for Doxorubicin Determination
Source: Biosensors (Basel). 2023 Apr 30;13(5):513. doi: 10.3390/bios13050513 (PMC10216684; doi:10.3390/bios13050513)
Supplement: Supplementary file 1 [file biosensors-13-00513-s001.zip › biosensors-2354600-supplementary.pdf]

Supporting Information

Impedimetric DNA Sensor Based on Electropolymerized N-phenylaminophenothiazine and Thiacalix [4]arene Tetraacids for Doxorubicin Determination

Tatiana Kulikova [1], Igor Shiabiev [1], Pavel Padnya [1], Alexey Rogov [2], Gennady Evtugyn [1,3], Ivan Stoikov[1] and Anna Porfireva [1,*]

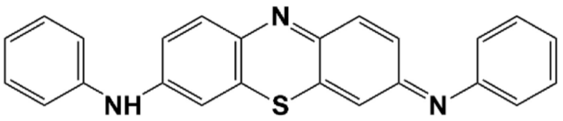

Figure S1. Chemical structure of *N*-Phenyl-3-(phenylimino)-3H-phenothiazin-7-amine studied.

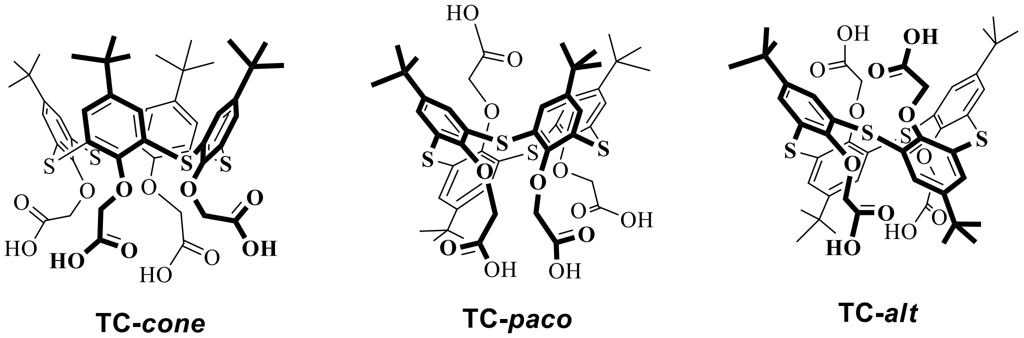

Figure S2. Chemical structures of thiacalix[4]arene tetracarboxylic acids studied.

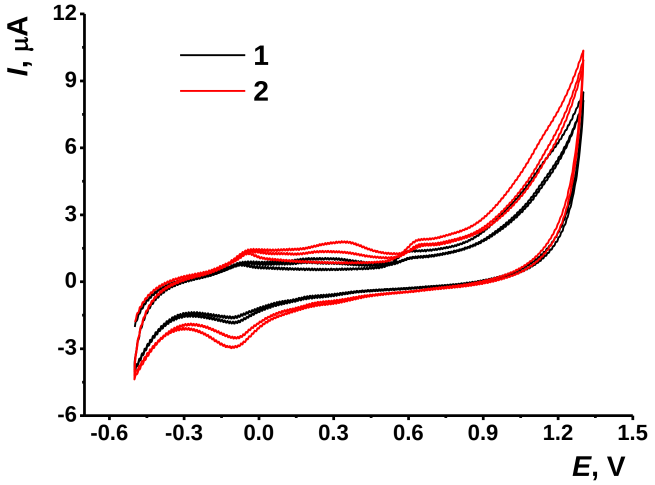

Figure S3. Cyclic voltammograms of PhTz polymerization recorded in phosphate buffer - acetone mixture (1:1 v/v) in the presence of 0.072 mM PhTz and TC-*cone* (1:2 molar ratio), scan rate 100 mV/s.
